# Abnormal Auditory Gain in Hyperacusis: Investigation with a Computational Model

**DOI:** 10.3389/fneur.2015.00157

**Published:** 2015-07-15

**Authors:** Peter U. Diehl, Roland Schaette

**Affiliations:** ^1^Bernstein Center for Computational Neuroscience, Berlin, Germany; ^2^UCL Ear Institute, London, UK

**Keywords:** hyperacusis, computational model, tinnitus, gain, auditory system

## Abstract

Hyperacusis is a frequent auditory disorder that is characterized by abnormal loudness perception where sounds of relatively normal volume are perceived as too loud or even painfully loud. As hyperacusis patients show decreased loudness discomfort levels (LDLs) and steeper loudness growth functions, it has been hypothesized that hyperacusis might be caused by an increase in neuronal response gain in the auditory system. Moreover, since about 85% of hyperacusis patients also experience tinnitus, the conditions might be caused by a common mechanism. However, the mechanisms that give rise to hyperacusis have remained unclear. Here, we have used a computational model of the auditory system to investigate candidate mechanisms for hyperacusis. Assuming that perceived loudness is proportional to the summed activity of all auditory nerve (AN) fibers, the model was tuned to reproduce normal loudness perception. We then evaluated a variety of potential hyperacusis gain mechanisms by determining their effects on model equal-loudness contours and comparing the results to the LDLs of hyperacusis patients with normal hearing thresholds. Hyperacusis was best accounted for by an increase in non-linear gain in the central auditory system. Good fits to the average patient LDLs were obtained for a general increase in gain that affected all frequency channels to the same degree, and also for a frequency-specific gain increase in the high-frequency range. Moreover, the gain needed to be applied after subtraction of spontaneous activity of the AN, which is in contrast to current theories of tinnitus generation based on amplification of spontaneous activity. Hyperacusis and tinnitus might therefore be caused by different changes in neuronal processing in the central auditory system.

## Introduction

Hyperacusis is a frequent auditory disorder that is characterized by an abnormal perception of loudness. Sounds that are comfortably loud to normal listeners are perceived as too loud or even painfully loud by people with hyperacusis, and a feeling of pain from sound is frequently described (1, 2). It is estimated that hyperacusis affects 2–15% of the population (3, 4). Hyperacusis might be closely related to the phantom auditory sensation of tinnitus, as it has been reported that about 85% of hyperacusis patients also have tinnitus (1, 5), and it is quite common for hyperacusis patients to develop tinnitus over time after the onset of hyperacusis. It has therefore been suggested that hyperacusis might be a precursor of tinnitus (6), and that both phenomena might be caused by similar mechanisms. However, only around 40% of the people with tinnitus also experience hyperacusis (4, 7), suggesting that hyperacusis could strongly facilitate the development or occurrence of tinnitus (8), whereas the presence of tinnitus might not contribute to hyperacusis.

Hyperacusis is characterized by abnormal perception of loudness. Several studies have therefore measured loudness discomfort levels (LDLs) and loudness growth functions of hyperacusis patients. LDLs were on average decreased by about 20 dB compared to normal-hearing subjects without sound-sensitivity problems (1, 5, 9, 10). Moreover, LDLs of hyperacusis patients have been found to be similar across the hearing range from 125 Hz to 8 kHz, suggesting a generalized increase in auditory gain or responsiveness (5). Interestingly, a recent study has reported that tinnitus subjects with normal hearing thresholds (HTs) also showed decreased LDLs (11), hinting at similarities between tinnitus and hyperacusis. However, an earlier study has shown that tinnitus patients without complaints of hyperacusis or sound-sensitivity problems have LDLs in the normal range (10), suggesting that the decrease of average LDLs reported in the former study for tinnitus subjects with normal HTs might have been caused by a subgroup with hyperacusis.

Loudness growth functions of subjects with hyperacusis have been investigated in two studies so far. Brandy and Lynn (12) investigated loudness growth in 25 hyperacusis subjects with normal hearing. Loudness growth was measured at 1 kHz only. Compared to the control group, the hyperacusis subjects exhibited a steeper slope of the loudness growth function, and sounds were rated as “too loud” already at lower sound intensities. Norena and Chery-Croze (13) reported on categorical loudness scaling results from eight subjects with hyperacusis and high-frequency hearing loss. Loudness growth was measured at three different frequencies, a frequency below the audiogram edge, the audiogram edge frequency, and a frequency in the region of hearing loss. Loudness growth functions were much steeper than normal at all three frequencies. Remarkably, even though loudness growth functions started at different sound levels at the three frequencies due to different degrees of hearing loss, the judgment category “too loud” was reached for approximately the same sound level in all three cases. A similar pattern of loudness growth at 500 and 2000 Hz has also been reported by Cox et al. (14) for a single patient. The loudness growth results of these studies thus suggest that hyperacusis is characterized by an increase in auditory gain or responsiveness that affects all sound intensities proportionally. It is also remarkable that the phenotype is qualitatively very similar in hyperacusis subjects with normal hearing (12) and with hearing loss (13).

The mechanisms that lead to the development of hyperacusis have remained obscure. A relation to hearing loss or cochlear damage has been suspected, but about a third of hyperacusis patients present with clinically normal HTs (1, 5). Therefore, if hyperacusis were caused by cochlear damage, only forms of cochlear damage that do not lead to an increase of HTs, i.e., hidden hearing loss (15, 16), might be considered as a potential trigger. This kind of cochlear damage can be caused by noise exposure (15, 17) or it can be age-related (18), with noise-induced damage occurring predominantly in the high-frequency range, and age-related damage equally along the length of the cochlea. It is now well established that cochlear damage can trigger neuronal plasticity in the central auditory system (19), and neuroplastic changes might also underlie the development of hyperacusis (20). Whether such changes might occur in hyperacusis in a frequency-specific manner, e.g., driven by the amount of cochlear damage in each part of the cochlea, or whether they occur in an unspecific manner through a generalized increase in sensitivity that affects all frequencies, has remained unclear.

Interesting data with respect to how gain mechanisms in the auditory system might operate have come from several recent studies on auditory deprivation through earplugs. Wearing an earplug for several days increases perceived loudness (21, 22), and decreases acoustic reflex thresholds (22, 23). Interestingly, when only one ear was plugged, perceived loudness was increased in both ears (22). When additional stimulation was provided, either through noise generators (21) or low-gain hearing aids (24), loudness was decreased and the acoustic reflex threshold increased. These studies indicate that the auditory system adapts to changes in the input it receives, and that loudness perception is modulated as a consequence. The observed changes in loudness and acoustic reflex threshold have been interpreted as changes in neuronal gain in the auditory system, and such gain changes might also underlie abnormal loudness perception in hyperacusis.

A recent theoretical study has postulated that hyperacusis might be due to an abnormal increase in neuronal gain in the central auditory system (25). Specifically, it was hypothesized that hyperacusis might be due to an increase in a non-linear gain mechanism, and that tinnitus on the other hand might be caused by an increase in “neuronal noise,” thus suggesting different mechanisms for the development of the two conditions. However, the predictions of the Zeng-model have not yet been evaluated through quantitative comparisons to data from hyperacusis patients. Moreover, recent computational modeling studies of tinnitus development have indicated that also tinnitus might be caused by an increase in responsiveness or gain of neurons in the central auditory system (26–30). In the tinnitus models, increased neuronal gain causes spontaneous neuronal activity to be amplified to such a degree that it crosses the perception threshold and thus gives rise to the phantom sound. As such an increase in neuronal gain will also cause amplification of sound-evoked activity, one might speculate that it could lead to a certain degree of hyperacusis. However, the gain increases in the tinnitus models are quite specific in that they first depend on the degree of hearing loss, and increased gain is thus confined to frequencies where hearing loss is present. Second, the gain increases in the tinnitus models amplify both spontaneous as well as evoked neuronal activity. Whether such a specific “tinnitus gain” can also explain the phenotype of hyperacusis seen in patient data remains to be determined.

In order to study the putative mechanisms of hyperacusis in more detail, and to derive predictions that can be compared to patient data, a detailed loudness model that also offers the possibility of incorporating various gain mechanisms would be desirable. The most commonly used loudness models are based on cochlear filterbank models of the basilar membrane [e.g., Ref. (31–33)]. The distribution of sound-evoked activity along the basilar membrane is called excitation pattern and forms the basis of those loudness perception models. Predictions about the loudness of a given sound are derived from the cochlear excitation pattern, which comprises the responses of all cochlear filters across frequencies. With the use of an appropriate basilar membrane model, the area under the cochlear excitation pattern is directly proportional to the loudness in sones (34). While such loudness models could in principle be used to study hyperacusis, the modeling of putative neural mechanisms of hyperacusis ideally requires more detailed simulations of neural activity. Even though the area of the cochlear excitation pattern in a filterbank model is related to auditory nerve (AN) activity levels, the relation might be complicated and non-linear, for example, due to response saturation of the different AN fiber types at different levels. A different approach would be to base a hyperacusis model on a more physiological cochlear model that also incorporates detailed simulations of AN fiber activity, like the Carney-model [e.g., Ref. (35)] or the Meddis-model [e.g., Ref. (36)]. Parameters to simulate the responses of a human cochlea, a pre-requisite for comparing model predictions to data from hyperacusis patients, are available for the latter modeling framework (37). However, so far, none of these detailed cochlear models have been tested for how well they account for loudness.

Here, we use a computational model that comprises the cochlea and AN as well as a “gain stage” (essentially a black box to incorporate a variety of gain mechanisms), to investigate which changes in auditory gain could account for the pattern of decreased LDLs typically seen in hyperacusis patients. Specifically, we investigate frequency-specific vs. frequency-independent changes in gain, linear vs. non-linear gain mechanisms, and mechanisms that work on spontaneous as well as evoked activity (similar to tinnitus models) vs. mechanisms that only amplify activity evoked by supra-threshold sounds, and all possible combinations of these features. For this purpose, we simulate AN responses using a model that is based on the MAP model by Ray Meddis and co-workers [reviewed in Ref. (36)]. In our model, the main assumption is that perceived loudness is proportional to the summed signal of all AN fibers across frequencies. The model parameters are first tuned such that the model reproduces loudness data from healthy normal hearing subjects. For the evaluation of putative hyperacusis gain mechanisms, we then focus on patients with normal HTs, since they constitute the largest subgroup of hyperacusis patients (1, 5). The model results suggest that decreased LDLs of hyperacusis patients are best accounted for by a generalized, frequency-independent increase in non-linear gain in the central auditory system. The gain is applied to supra-threshold responses only, i.e., after subtracting the spontaneous activity of the AN. If also the spontaneous activity of the AN is amplified, as in computational models of tinnitus development, or if an increase in linear gain is applied, the model predictions are not consistent with the data.

## Materials and Methods

### LDL and hearing threshold data

Two different data sets were used to fit our model. The first one consisted of the audiograms and LDLs of 13 control subjects. HTs were measured at 0.25, 0.5, 1, 2, 4, and 8 kHz, and LDLs at 0.5, 1, 2, and 4 kHz. These data were collected in a previous study at the UCL Ear Institute, which had been approved by the UCL research ethics committee. The second data set comprised 130 patients with a primary complaint of hyperacusis and normal HTs (≤20 dB HL, where dB HL is the sound level, which is normalized such that the average person’s HT is 0), who were treated at the Tinnitus and Hyperacusis Centre in London. The patient data set is a subset of the hyperacusis patient data presented in Sheldrake et al. (5). For all patients, HTs and LDLs had been measured at 0.125, 0.25, 0.5, 1, 2, 4, and 8 kHz. This data set was collected as part of the routine intake examinations for hyperacusis patients at the London Tinnitus and Hyperacusis Centre, and has been made available to us in anonymized form.

**Figure 1:**
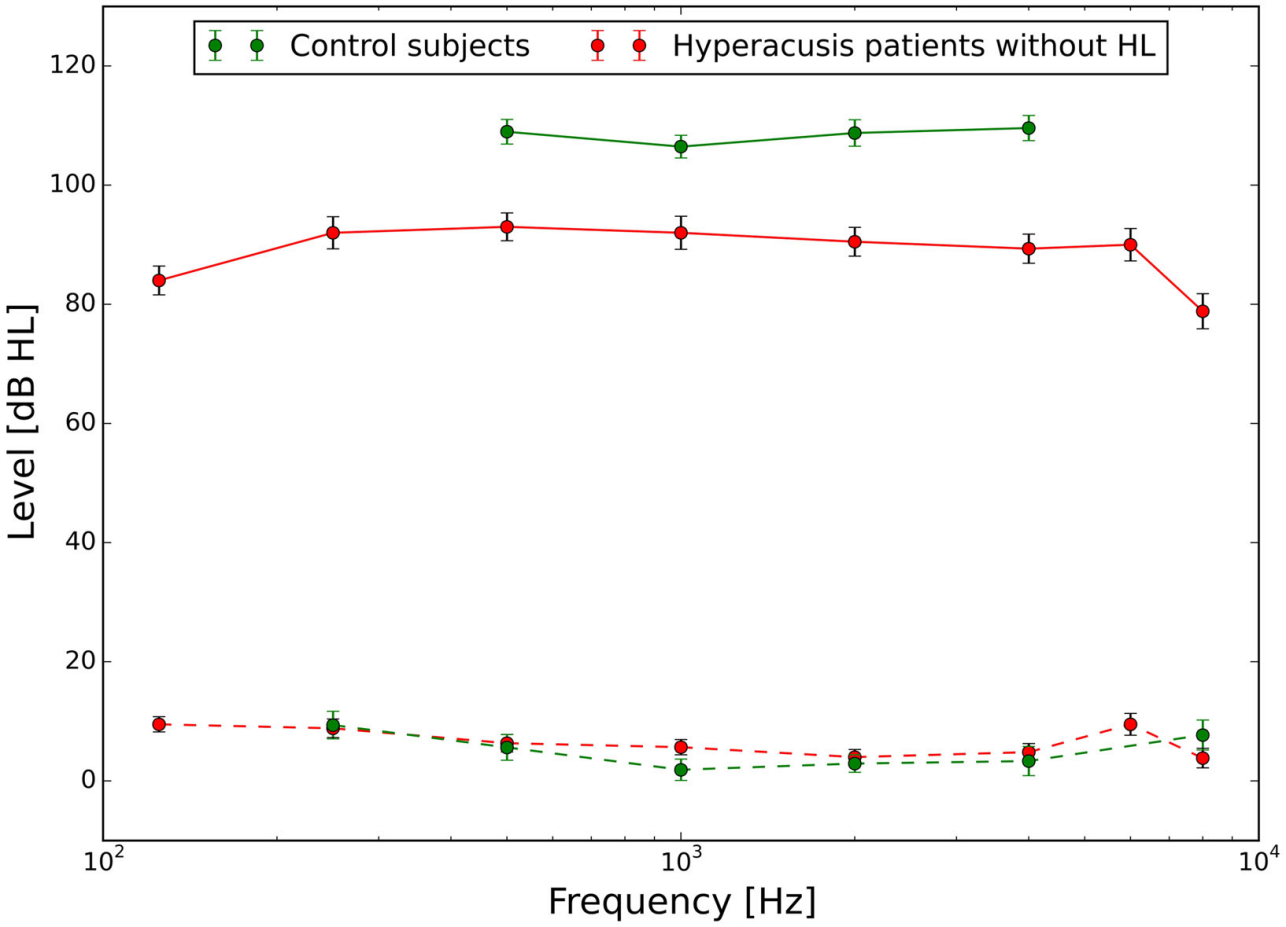
**Hearing thresholds (dashed lines) and loudness discomfort levels (LDLs, solid lines) of normal-hearing control subjects (green) and hyperacusis patients (red)**. Hyperacusis patient data are from a subgroup of patients with clinically normal HTs, the complete patient data set is presented in Sheldrake et al. (4).

The mean audiograms and loudness discomfort levels of the hyperacusis and the control group are shown in Figure 1.

### Model

**Figure 2:**
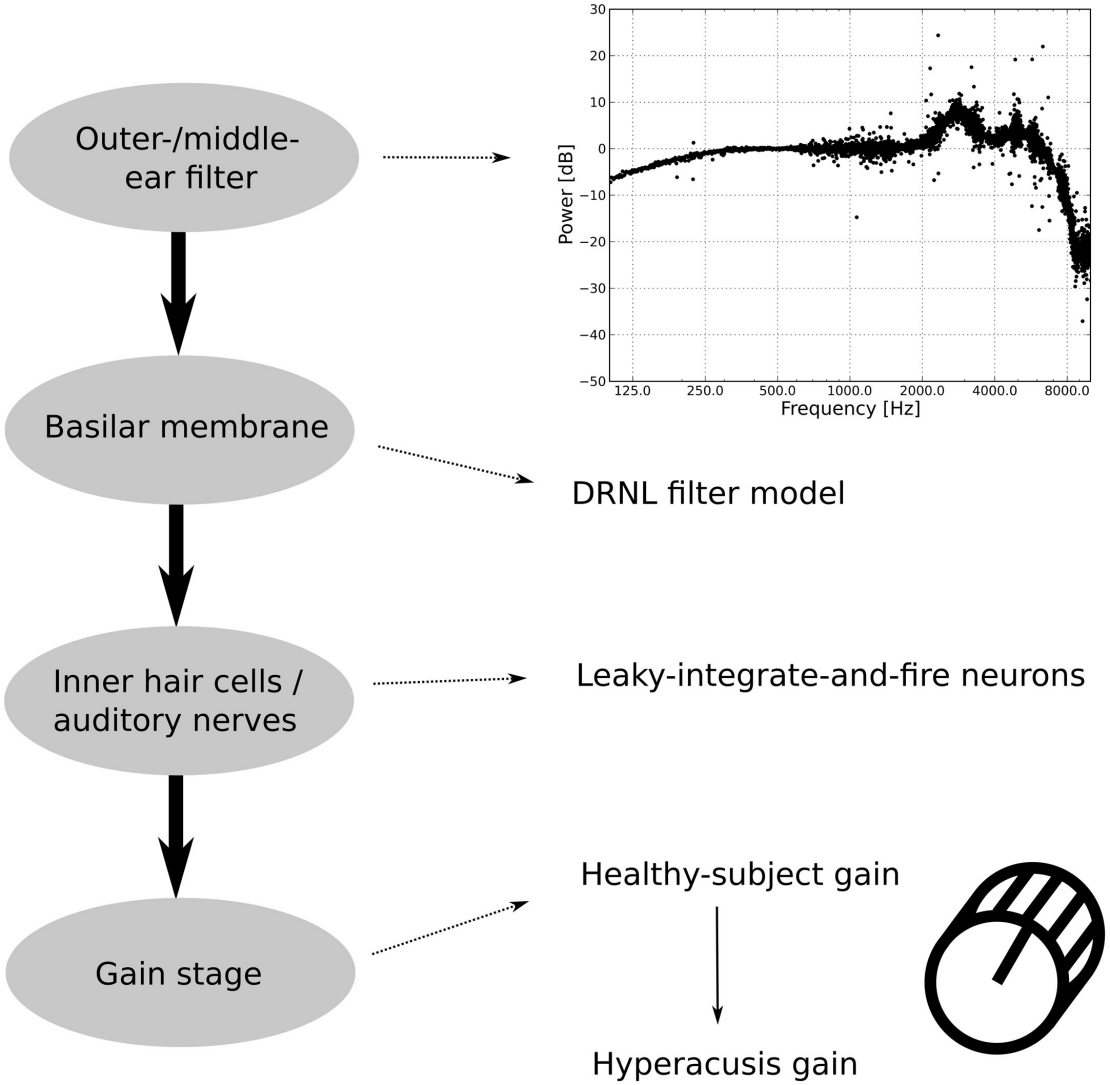
**Structure of the auditory model**. The outer/middle ear filter consists of multiple bandpass filters. The difference in the power spectrum between an outer/middle ear-filtered white-noise stimulus and an unfiltered white-noise stimulus is displayed in the upper graph. The second stage, the basilar membrane model, is the DRNL filter model. The output of the basilar membrane model is fed into leaky-integrate-and-fire neurons, which model the inner hair cells and the auditory nerves. The last part of the auditory model is the gain stage, where first a healthy-subject gain is applied to fit healthy-subject data and then a hyperacusis gain.

The model is based on the model of the auditory periphery by Meddis and co-workers (36–39), and it comprises four different stages (see Figure 2). Input sounds are first fed into an outer/middle ear filter, which converts sound pressure into stapes velocity. The next step is a basilar membrane model, which in turn feeds its output to a spiking neuron model that approximates inner hair cells (IHC) and AN fibers. In the last processing step, the AN signal is fed into a gain stage, which is used to incorporate different candidate mechanisms for a “hyperacusis gain.” Predictions of perceived loudness are then based on the output of the gain stage.

In the basic, “healthy” model, the gain stage comprises only one sub-module, which is used to tune the model to reproduce normal loudness perception. To model hyperacusis, a second sub-module is added to the gain stage, with the second gain being applied after the first one.

The model was implemented using the neural simulator BRIAN (40) and the BRIAN.Hears package (41), which are written in the programing language Python. All sound stimuli had a duration of 10 s in our simulations.

#### Outer and Middle Ear Model

The first module of the model is the outer and middle ear filter, which has been adapted from version 1.7 of the model implementation of Meddis (38). The filter consists of three parallel bandpass Butterworth filters with lower cut-off frequencies of 1900, 4500, and 8000 Hz, upper cut-off frequencies of 4200, 6300, and 12,000 Hz, and gains of −2, −3, and −19 dB, respectively. The upper graph in Figure 2 shows the difference in the power spectrum of a white-noise stimulus before and after filtering by the outer/middle ear filter. The details of the filter are described in Lopez-Poveda et al. (37).

#### Basilar Membrane Model

To model basilar membrane responses, we have employed the dual resonance non-linear (DRNL) filterbank model (36–39), which is widely accepted as a model that reproduces salient properties of the basilar membrane response to sounds. Specifically, we have used it with the parameter set for a human cochlear filterbank, which has been developed by Lopez-Poveda and Meddis (37).

A DRNL filter approximates the response of the basilar membrane at a certain place of the cochlea, which is linear at very low sound intensities, compressive at medium sound intensities, and then linear again for very high sound intensities. To achieve this, each filter of the DRNL comprises a linear and a non-linear pathway. The linear pathway consists of a linear gain, two first-order gammatone filters and four second-order Butterworth low-pass filters. The non-linear pathway consists of three first-order gammatone filters, a broken-stick non-linearity, three first-order gammatone filters, and three second-order Butterworth low-pass filters. The output of the broken-stick non-linearity *y(t)* at time point *t* depends on the parameters *a*, *b*, and *c* and on the input *i(t)* (37):

$$y\left(t\right)=\text{sign}\left[i\left(t\right)\right]\times \text{min}\left[a|i\left(t\right)|,\;\;b|i\left(t\right){|}^{\text{c}}\right]$$

Here, the input *i(t)* is the peak stapes velocity in meters per second, pre-processed by the three first-order gammatone filters. This broken-stick non-linearity is constructed such that the parameter *a* governs the amplification of soft sounds, and the parameters *b* and *c* determine the point at which the basilar membrane response becomes compressive and the degree of compression, respectively.

In the last step, the outputs of both pathways are summed and multiplied by a constant converting the output to the basilar membrane velocity in meter per second. The parameters of both pathways are chosen such that the non-linear pathway is dominating for low input intensities and the linear pathway is domination for high input intensities, and they vary systematically with the characteristic frequency of the DRNL filter.

In our implementation of the DRNL model, we have used 500 filters with center frequencies between 40 Hz and 13 kHz, which were distributed evenly on an ERB-scale (42). We have used the implementation of the DRNL filter bank included in the BRIAN.Hears package. However, as this implementation differed slightly from the model of Lopez-Poveda and Meddis (37), we first changed the parameter “g0” from −0.48 to 0.48 [the value used in Ref. (37)], and we used three gammatone filters as in Ref. (37), instead of two. Moreover, we added an outer and middle ear model (see above), which was not contained in the BRIAN.hears package (43).

#### Inner Hair Cell and Auditory Nerve Model

In our model implementation, the outputs of the DRNL filters was fed as input currents into leaky-integrate-and-fire (LIF) neurons, which are used to model IHC and AN fibers in a simplified fashion. The output of every single one of the 500 filters was fed into three LIF neurons with different activation thresholds, i.e., we have used 1,500 LIF neurons where groups of three LIF neurons receive input from the same DRNL filter. To model the different levels of spontaneous activity of the three types of AN fibers, the three groups of neurons also received a noise current at different levels, which leads to different spontaneous firing rates. Note that this is a strong abstraction from the actual physiological process, which was chosen to simplify simulations, as the detailed temporal response patterns of AN fibers are not relevant for our study. A much more detailed model of this apparatus is presented in Meddis (38). The parameters of the LIF neurons (threshold and noise current) were adjusted such that the resulting response characteristics corresponded to the three different types of AN fibers [e.g., Ref. (44)]. Similar to the model of Sumner et al. (45), we generated low-threshold fibers with an average spontaneous rate (SR) of 35 sp/s, medium-threshold fibers with an SR of 8 sp/s, and high-threshold fibers with SR of 2 sp/s.

#### Calculation of Loudness in Sones

The sone is a unit of loudness. A 1-kHz tone at 40 dB SPL is defined as having the loudness of 1 sone, and a doubling of the perceived loudness corresponds to a doubling of the sone value. In our model, the perceived loudness of a tone in sones is calculated from AN activity. More specifically, it is based on the summed activity of all AN fibers in response to the presentation of a tone, using the formula

$$\text{Ldn}(f,L)={\left(\frac{\sum_{\text{cf}}R_{\text{AN}}(f,L,\text{cf})-R_{{\text{AN}}_{\text{sp}}}}{\sum_{\text{cf}}R_{\text{AN}}(1\text{kHz,40dB,cf})-R_{{\text{AN}}_{\text{sp}}}}\right)}^{\text{x}}$$

where the stimulus is a tone at frequency *f* and level *L*, _Σcf_*R*_AN_(tone, cf) is the sum of AN responses elicited by the stimulus, _Σcf_*R*_AN_(1 kHz, 40 dB, cf) is the sum of the firing rate responses of the AN fibers elicited by a 1-kHz tone at 40 dB SPL, and *R*_ANsp_ is the sum of the spontaneous firing rates of all AN fibers (measured in the model by stimulating with a 1-kHz tone at −10 dB SPL). The denominator _Σcf_*R*_AN_(1 kHz, 40 dB, cf) − *R*_ANsp_ ensures that a 1-kHz-tone at 40 dB SPL produces a loudness of 1 sone in the model. The exponent *x* was adjusted to match the loudness predicted from AN responses to the ANSI S3.4-2007 standard. Fitting was done by minimizing the mean squared error, and the resulting value was *x* = 1.61.

#### Equal-Loudness Contours

Our main assumption for deriving equal-loudness contours (ELCs) from the model is that two tones that produce the same level of overall AN activity will be perceived as equally loud. We determined ELCs in reference to 1 kHz tones, similar to determining perceived loudness in units of phon, where a sound is defined to have the loudness of *x* phon when it is perceived as loud as a 1-kHz tone at *x* dB SPL. ELCs were determined for 1 kHz tones ranging from −10 to 130 dB SPL in 10 dB steps, and additionally also for 1 kHz tones at −5, −2, and 2 dB SPL.

Deriving ELCs for the model was thus a two-step process. We first determined the summed AN firing rate response _Σcf_*R*_AN_(1 kHz, *L*, cf) for the 1-kHz tones at all levels. For all other tone frequencies (0.125, 0.25, 0.5, 2, 3, 4, 6, and 8 kHz), we then searched for the stimulus intensities that evoked the same total AN firing rate response as the 1-kHz reference tone, repeating the process for each level of the 1-kHz reference tone, yielding a model ELC for each level of the 1-kHz reference tone. To restrict the simulation time required for this, we simulated AN responses to tones with a variable intensity step size (2-dB steps close to the HT, and 10 dB steps above 10 dB HL), and interpolated the AN response linearly between sound levels if the target AN response was between two levels.

#### Gain Stage

As outlined above, model predictions of perceived loudness are based on the summed activity of all AN fibers in response to the stimulus. The simplest way to change predicted loudness is thus to apply some gain to this signal and amplify or dampen it. Since we were mainly interested in which gain mechanisms would lead to a change of perceived loudness consistent with the pattern of reduced LDLs seen in hyperacusis patients, we used a simple gain stage in the model to evaluate the different candidate mechanisms.

In a first step, the gain stage of the model was used to tune the model to reproduce normal loudness perception by matching model predictions to the LDLs of healthy control subjects. The gain change required for this is thus called “healthy control gain.” For modeling hyperacusis, we applied a second gain after the healthy control gain, the “hyperacusis gain.” While the healthy control gain was the same for all models, we tried eight different hyperacusis gains (see Figure 2).

##### Healthy control gain

To tune the model for normal loudness perception, the gain stage contains a frequency-dependent gain (Figure 2), i.e., an individual gain factor is applied to the firing rate response of each AN fiber according to its characteristic frequency. The gain was hand-fitted such that the model LDLs were similar to those of our control subjects. This was achieved by fitting a frequency-dependent gain *g*_hc_(cf), which weights the output of every single AN fiber, and therefore the contribution of each single AN fiber to perceived loudness. In order to prevent over-fitting of the model, we restricted the shape of the frequency dependence to quadratic functions:

$$g_{\text{hc}}\left(\text{cf}\right)=a+b\times {\left(\text{cf}-c\right)}^{2}$$

where *cf* is the characteristic frequency of an AN fiber and *a, b*, and *c* are the parameters, which are fitted to match the model to data from the control subjects.

##### Linear vs. power-law hyperacusis gain

One possibility of how to apply the hyperacusis gain is in a linear fashion, i.e., the resulting neural activity *R*_l_ is the firing rate response of each AN fiber is multiplied by a gain factor *g*_l_(cf):

$$R_{\text{l}}\left(f,\;L,\text{ cf}\right)=g_{\text{l}}\left(\text{cf}\right)g_{\text{hc}}\left(\text{cf}\right)R_{\text{AN}}\left(f,\;L,\text{ cf}\right)$$

where *R*_AN_(*f*, *L*, cf) is the firing rate response of the AN fiber with characteristic frequency *cf* to a tone at frequency *f* and level *L*, and *g*_hc_(cf) the corresponding healthy control gain factor.

The other possibility we considered is that the hyperacusis gain depends on the firing rate of the AN fiber. Specifically, the dependence of the gain factor *g*_p_ on AN fiber activity takes the form of a power-law function:

$$g_{\text{p}}\left(f,\;L,\text{ cf}\right)={\left(\frac{R_{\text{AN}}\left(f,\;L,\text{ cf}\right)}{R_{\text{ANmax}}\left(\text{cf}\right)}\right)}^{\text{z}}g_{\max}\left(\text{cf}\right)$$

where *R*_AN_(*f*, *L*, cf) is the firing rate response of the AN fiber with characteristic frequency *cf* to a tone at frequency *f* and level *L*, *R*_ANmax_(cf) is the maximum firing rate of the AN fiber, and *g*_max_(cf) is the maximum value of the gain factor, which may also depend on the frequency (see below). The exponent *z* was hand-fitted such that the loudness growth for low-intensity tones was proportional to the loudness growth of high-intensity tones. The resulting value of the exponent *z* was 1.1, and it was kept constant for all hyperacusis simulations, whereas the value of *g*_max_(cf) was varied to fit the model to the data. Note that since the base under *z* is always ≤1 (because of the normalization by the maximum firing rate), the resulting values of *g*_p_(*f*, *L*, cf) are always ≤*g*_max_. The neural activity *R*_p_ after application of the power-law gain is then

$$R_{\text{p}}\left(f,L,\text{cf}\right)=g_{\text{p}}\left(f,L,\text{cf}\right)g_{\text{hc}}\left(\text{cf}\right)R_{\text{AN}}\left(f,L,\text{cf}\right).$$

##### Sub-threshold vs. supra-threshold hyperacusis gain

The gain can either be applied only to sound-evoked firing rate responses of the AN fibers, i.e., for firing rate responses that exceed the SR, which we will call supra-threshold gain, or to all AN activity including spontaneous firing, which we will call sub-threshold gain. The sub-threshold gain is thus simply applied by multiplying the firing rate of each AN fiber with the corresponding gain factor. The neural activity *R*_sup_ after application of the supra-threshold gain mechanism is calculated using

$$R_{\text{sup}}\left(f,L,\;\text{cf}\right)=\left(\text{max}\left(0,\;R_{\text{AN}}\left(f,L,\text{cf}\right)-R_{\text{ANsp}}\left(\text{cf}\right)\right)\times g+\;R_{\text{ANsp}}\left(\text{cf}\right)\right)g_{\text{hc}}\left(\text{cf}\right)$$

where *R*_ANsp_(cf) is the spontaneous firing rate of the AN fiber and *g* the corresponding hyperacusis gain factor, which may be a linear or a power-law gain factor. Note that since the noise current used in the LIF neurons to generate spontaneous AN fiber activity differs from simulation to simulation, it is possible that the AN fiber responses for very soft tones can be below the average spontaneous activity. In this case, the supra-threshold gain would not be applied at all to the response of the corresponding fibers.

##### Frequency-dependent vs. frequency-independent hyperacusis gain

Finally, we consider frequency-dependent as well as frequency-independent changes in gain. For the case of frequency-independent gain changes, we simply used constant values, i.e., *g*_l_(cf) = *g*_l_ and *g*_max_(cf) = *g*_max_ that were applied to all frequency channels in the model.

For frequency-dependent gain changes, we constrained the degrees of freedom of the model by allowing for *g*_l_(cf) and *g*_max_(cf) only gain changes that followed a quadratic function, similar to the healthy-subject gain (see above):

$$g\left(\text{cf}\right)=a+b\times {\left(\text{cf}-c\right)}^{2}$$

The three free parameters of the equation were hand-fitted such that the ELCs predicted by the model were as close as possible to the hyperacusis patient data.

## Results

### Model properties

The goal of this study was to investigate which changes in gain in the central auditory system can account for uniformly decreased loudness discomfort levels as seen in hyperacusis patients (Figure 1). As a first step, we therefore tuned the “healthy ear variant” of the model such that it correctly predicted the loudness of a sound in sones, and also produced ELCs that were in accord with HTs and LDL values of normal-hearing control subjects. The main assumption for this task was that perceived loudness is proportional to the summed activity of all AN fibers.

**Figure 3:**
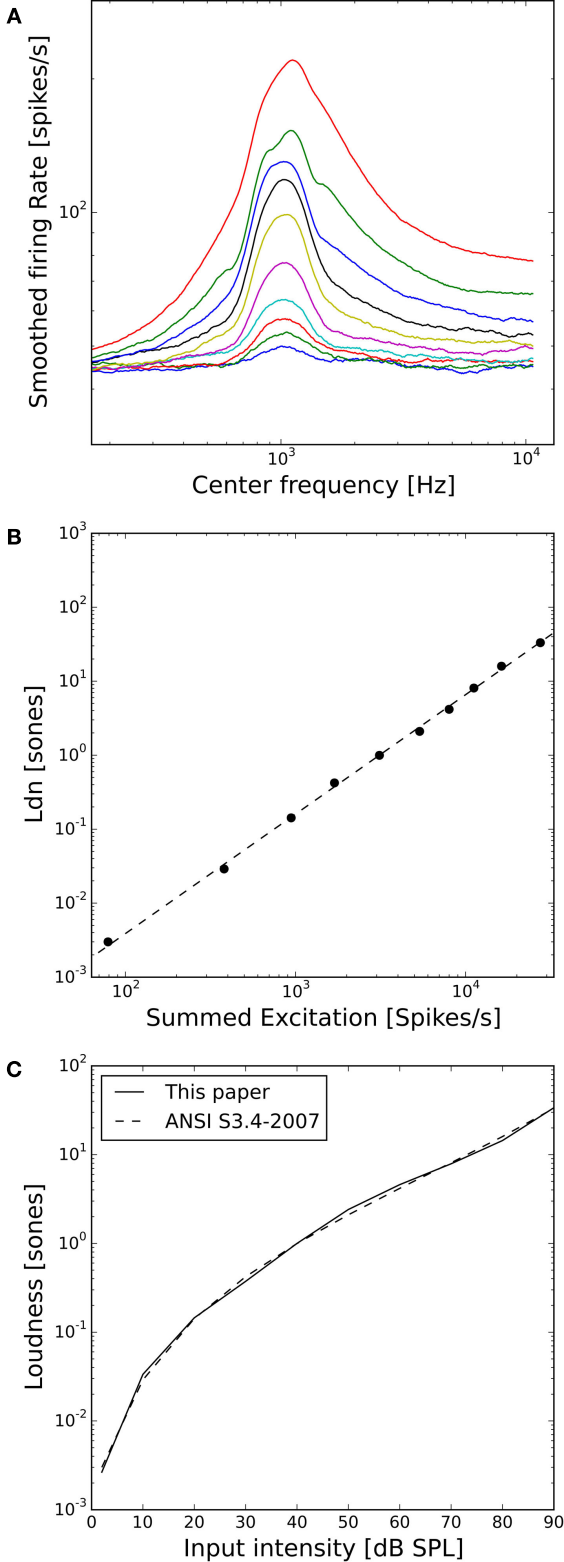
**Loudness model based on simulations of auditory nerve fiber activity**. **(A)** Model auditory nerve activity patterns for 1 kHz tones. Tones were presented at 2 dB SPL and then from 10 to 90 dB SPL in 10 dB steps. The AN fiber activity patterns have been smoothed using a moving average over 50 AN fibers. **(B)** Relation between the summed activities of all model AN fibers and loudness in sones according to ANSI S3.4-2007 (46). The dashed line shows a fitted power-law function with a slope of 1.1. **(C)** Loudness growth function of our model compared to the ANSI S3.4-2007 (46) standard.

Figure 3A shows the activity of the whole AN population of our model in response to 1 kHz tones of 10–90 dB SPL. The distribution of activity across frequencies is qualitatively very similar to the cochlear excitation patterns of phenomenological loudness models. Moreover, the summed activity of the AN fibers in response to the 1 kHz tones (after subtracting the spontaneous activity and applying a power-law function, see Materials and Methods) turned out to be directly proportional to the loudness of the stimuli in sones according to ANSI S3.4-2007 (Figure 3B), and the predicted loudness growth function closely matched the standard (Figure 3C).

**Figure 4:**
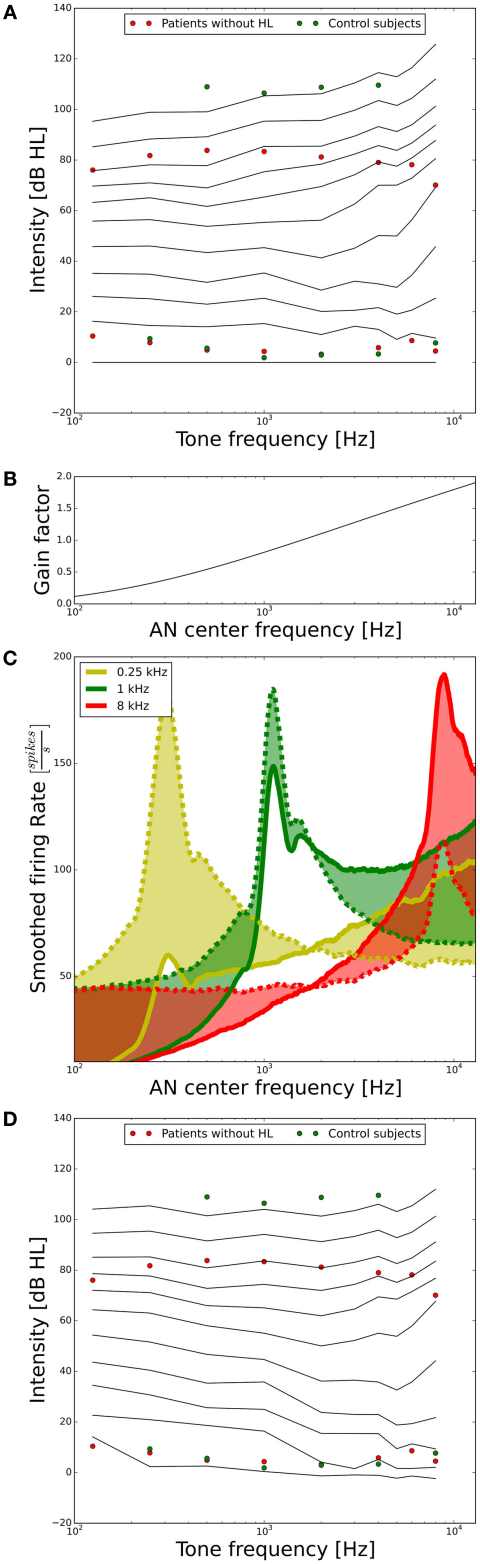
**Tuning the model to fit hearing threshold and LDL data from normal-hearing control subjects**. **(A)** Equal-loudness contours of the model in dB HL (black lines) before parameter tuning. The circles indicate HTs and LDLs of the control subjects (green) and the hyperacusis patients (red). **(B)** Frequency-dependent gain factor fitted to tune the model, “healthy gain.” **(C)** Illustration of the effect of the healthy gain factor on the model activity profile for stimulation with 0.25 (yellow), 1 (green), and 8 kHz tones (red) at 70 dB HL. The dashed lines show the activity profile before, and the solid lines after application of the gain factor. The shaded areas emphasize the difference. Note that the activity profiles have been smoothed by applying a running average over 25 AN fibers. **(D)** Equal-loudness contours after applying the “healthy control gain” factor to the auditory nerve responses.

As a next step, we assessed the ELCs predicted by our model. For our purposes, it will suffice if the ELCs are qualitatively similar to the standard ISO 226-2003 (2003), since there are many factors, which can influence ELCs, for example, if the data are recorded in free-field or diffuse-field condition, or the type of headphones used. A plot showing the ELCs of our model, where the *y*-axis is normalized to “model” dB hearing level (HL) is shown in Figure 4A. To obtain the ELCs in “model dB HL”, the sound levels of the 2-phon-ELC (obtained by determining the sound intensity required to produce the same summed AN fiber response as a 1-kHz tone at 2 dB SPL, see Materials and Methods) were subtracted from all other ELCs at all frequencies, since the 2-phon-contour roughly corresponds to the HT (34). The green dots in Figure 4A show average HTs and average LDLs of the control subjects. Red dots depict average HTs and average LDLs of hyperacusis patients with normal hearing thresholds from the clinical data set. Those HTs and LDLs are included in the plot to provide a comparison of our model results to empirical data. After the transformation to “model HL” (see above), the resulting model predictions of ELCs for high sound intensities were still very different from the LDLs of control subjects. Low-frequency tones were predicted as too loud, and high-frequency tones as too soft (Figure 4A). To address this, we used the gain stage of our model, applying a linear gain factor for every AN fiber as explained in the section “Materials and Methods.” This linear frequency-dependent gain was then fitted to match the psychophysical data of the control subjects; in the following, we will refer to this gain as the “healthy control gain.” The resulting values of the gain factor are shown in Figure 4B, the change of the responses of the AN fibers is shown in Figure 4C, and the resulting ELCs after application of this gain are shown in Figure 4D. With the “healthy control gain,” the ELCs at high sound intensities were parallel to the LDL curve of the control subjects, and interestingly also the match for the HTs was improved. Using this model of normal loudness perception, we then investigated which changes in auditory gain might lead to hyperacusis.

### Evaluation of different gain mechanisms to model hyperacusis

Our main goal was to investigate which gain mechanisms or modifications could explain hyperacusis, i.e., which changes could shift ELCs in the model to such a degree that the ELC corresponding to the LDLs in the healthy case matched the average LDL curve of hyperacusis patients. To induce such an increase of the perceived loudness, we used many different types of potential “hyperacusis gains.” First, either a linear gain, which acts on the output of the AN fibers independent of their activity, or a power-law gain, where the gain depends on AN activity in that low activity levels are amplified less than high activity levels (see Materials and Methods). Second, either a gain that varies according to the characteristic frequency of the AN fibers (“frequency-dependent”), or a “frequency-independent” gain where the same gain factor is applied to all frequency channels. Lastly, the gain was either applied to all of the AN activity including the spontaneous activity (“sub-threshold”), or only to evoked responses that exceeded the level of spontaneous activity (“supra-threshold”).

**Figure 5:**
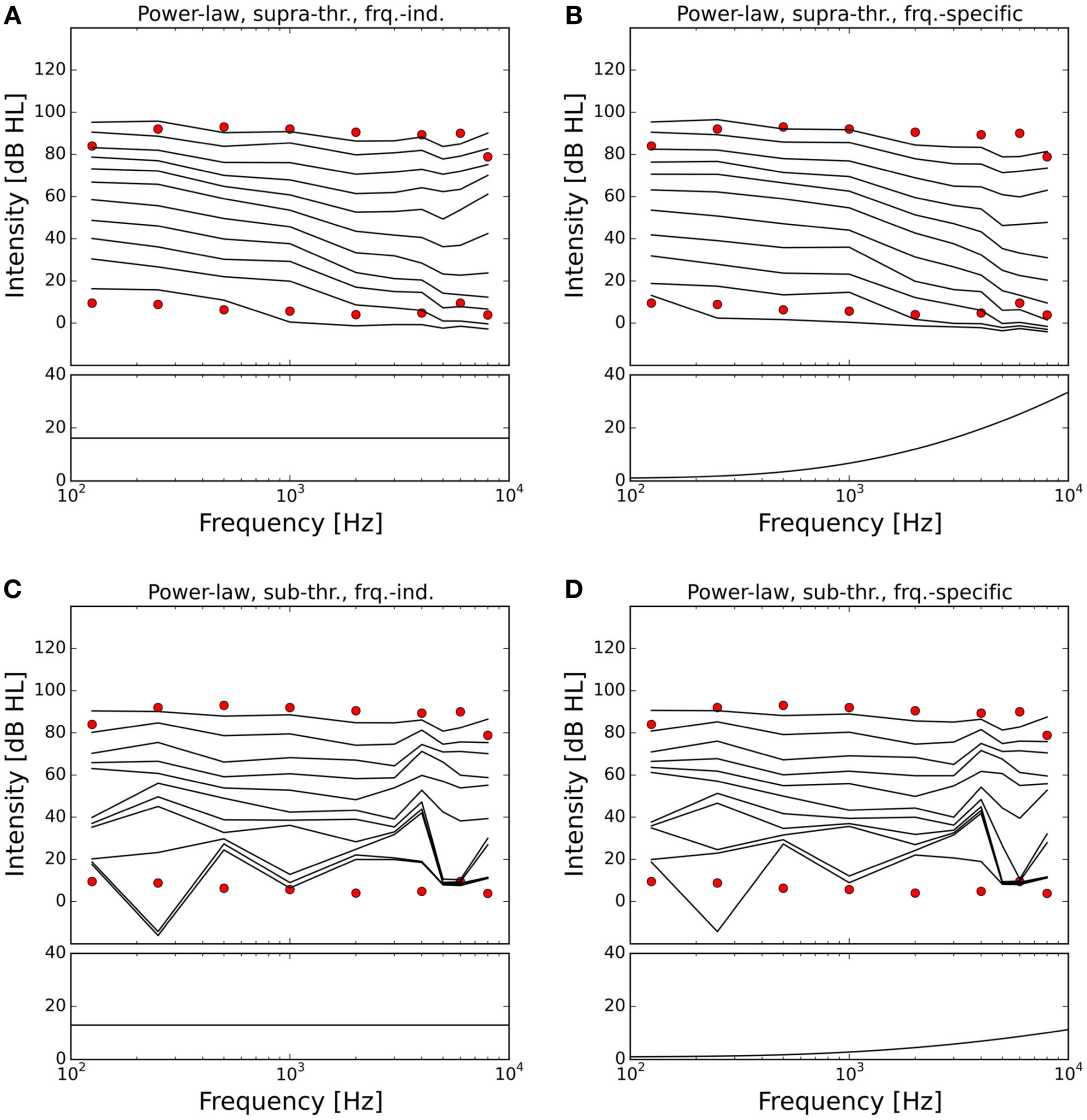
**Modeling hyperacusis with power-law gain**. Equal-loudness contours are shown in the top panels, fitted gain factors in the bottom panels. Note that the gain factors have been applied in addition to the healthy control gain. **(A)** Supra-threshold frequency-independent power-law gain: the model equal-loudness contours are in good agreement with the LDLs of the hyperacusis patients and show steeper loudness growth across the intensity range. **(B)** Supra-threshold frequency-specific gain: gain increase predominantly in the high-frequency range also produced a good match to hyperacusis patient data. **(C)** Sub-threshold frequency-independent gain: modification of the spontaneous activity of the AN fibers leads to distorted HTs. **(D)** Sub-threshold frequency-specific gain: as in **(C)**.

**Figure 6:**
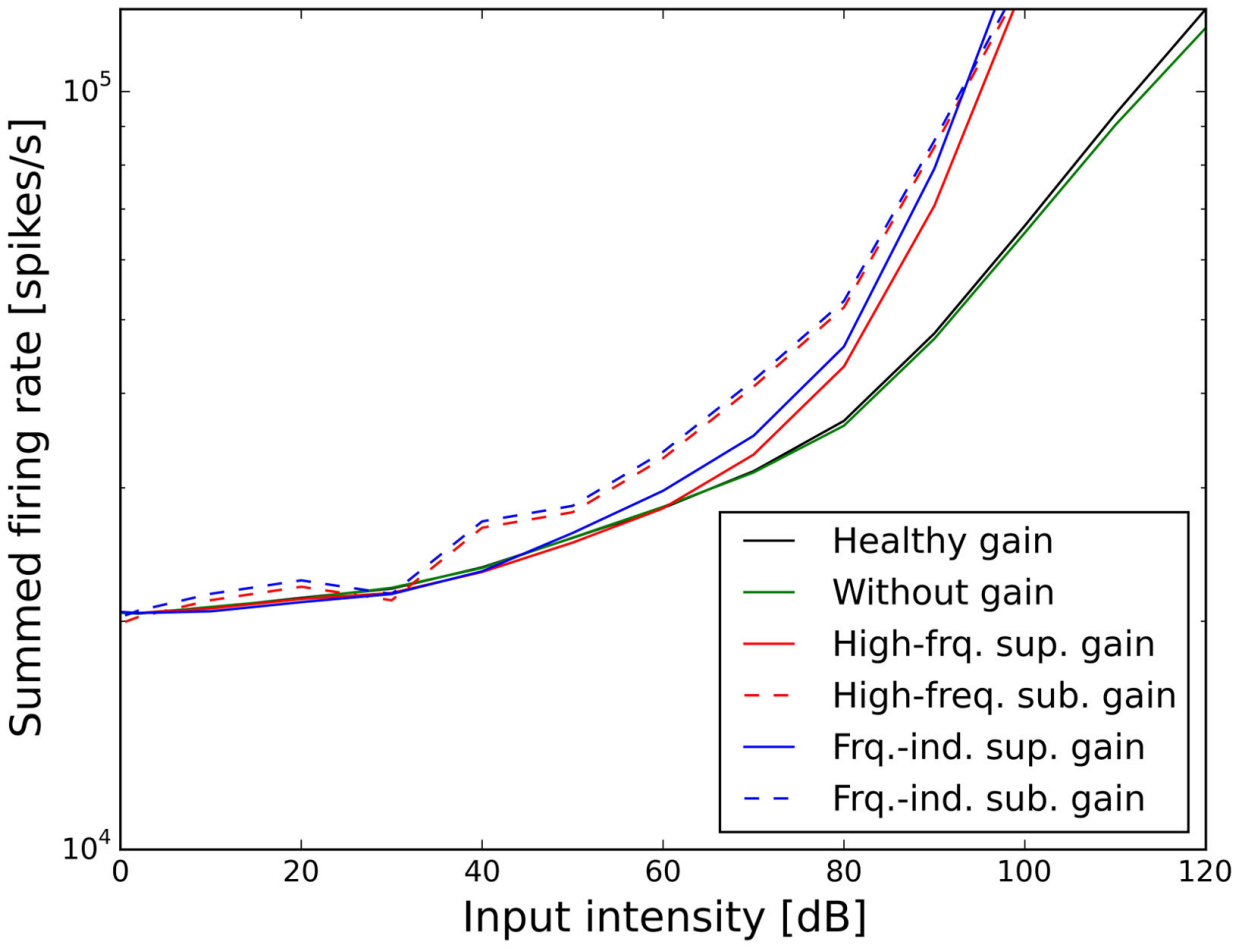
**Effect of the different scenarios with power-law gain change on total neural activity in the model**. Results for stimulation with 1 kHz tones are shown.

The resulting ELCs for all possible combinations of a power-law gain with supra-threshold or sub-threshold gain and frequency-independent or frequency-specific gain are depicted in Figure 5, and the corresponding growth functions of neural activity in the model are shown in Figure 6. The best fit of the LDL curve of the hyperacusis patients was achieved using a supra-threshold frequency-independent power-law gain (Figure 5A). After application of this gain, the model ELC that corresponded to the LDL in the healthy situation was shifted to lower intensities, with a very good match to the average hyperacusis LDLs in the frequency range from 250 Hz to 6 kHz. At 125 Hz and 8 kHz, the model predicted LDLs slightly too high. The activity growth curve showed a strong increase in activity only for higher intensities (Figure 6), reminiscent of a multiplicative gain (25). This growth function was very similar to the one we achieved using a frequency-specific supra-threshold power-law gain, which also provided a good fit to the hyperacusis patient data (Figure 5B). The model with the frequency-specific supra-threshold power-law gain was able to match the patient LDLs from 250 Hz to about 2 kHz, and then again at 8 kHz. LDLs for 4 and 6 kHz were predicted as slightly too low, and at 125 Hz as too high. Overall, both variants of the supra-threshold non-linear gain provided a decent match to the data, which could have been further improved by allowing more degrees of freedom for the function governing the frequency-dependence of the gain, which we had limited to quadratic functions (see Materials and Methods).

In contrast to this, both sub-threshold power-law gains showed artifacts in the ELCs (Figures 5C,D) and the loudness growth curves (dashed lines in Figure 6) for low sound intensities. The reason for these artifacts is that for very low input intensities and thus AN activity levels, the power-law gain may decrease the AN fiber activity such that it ends up below the normal level of spontaneous activity. As a consequence, the model HT then depends on amplification of even slight variations of spontaneous or sound-evoked AN firing rate responses, since AN fiber activity is most variable at low sound intensities. This amplification of response fluctuations at low sound intensities is an inherent property of the sub-threshold power-law gain, which then causes the pronounced differences in HTs for different frequencies. For the results presented here, it would be possible to average out those noise-induced differences by averaging over longer sound presentations, or to increase the number of AN fibers, since the number of AN fibers in our model is an order of magnitude lower than in the human ear. However, the stimulus duration of 10 s used in our simulations is already about an order of magnitude longer than typical stimulus durations in loudness measurements, thus compensating for the low number of AN fibers. The artifacts at low sound intensities therefore reflect a limitation of the sub-threshold power-law gain, which is not present in the case of the supra-threshold power-law gain.

**Figure 7:**
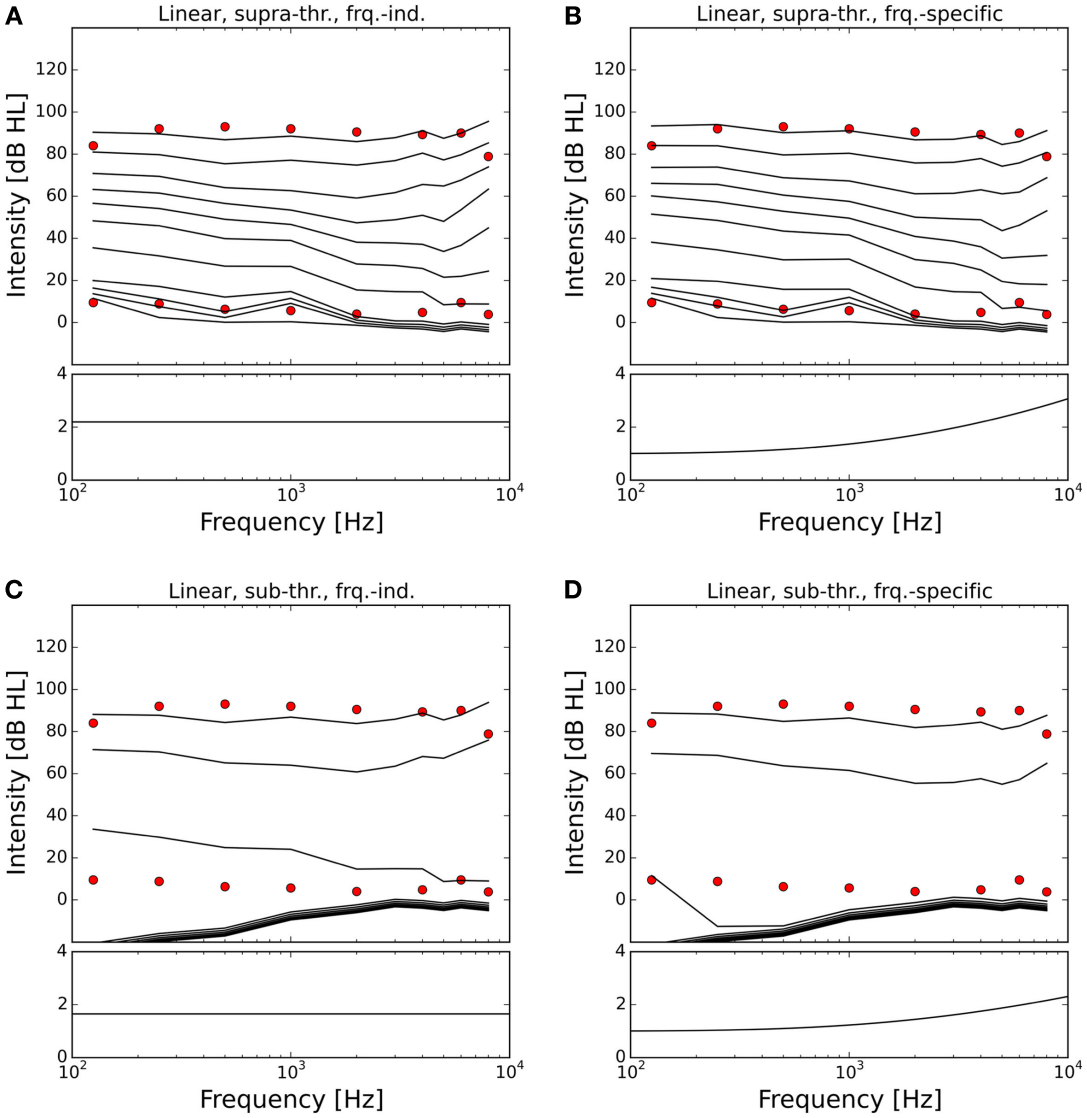
**Modeling hyperacusis with linear gain**. Equal-loudness contours are shown in the top panels, fitted gain factors in the bottom panels. Note that the gain factors have been applied in addition to the healthy control gain. **(A)** Supra-threshold frequency-independent gain: the equal-loudness contours show an abrupt, almost step-like increase of loudness near threshold, and a good match to patient LDLs. **(B)** Supra-threshold frequency-specific gain: gain increase in the high-frequency channels also provided a good match to patient LDLs, but again with the abrupt, step-like increase of loudness near threshold. **(C)** Sub-threshold frequency-independent gain produces a constant broad-band tinnitus at 70–80 phon when LDLs are matched to patient data. **(D)** Sub-threshold frequency-specific gain produces a high-frequency tinnitus at 80 phon when LDLs are matched to patient data.

**Figure 8:**
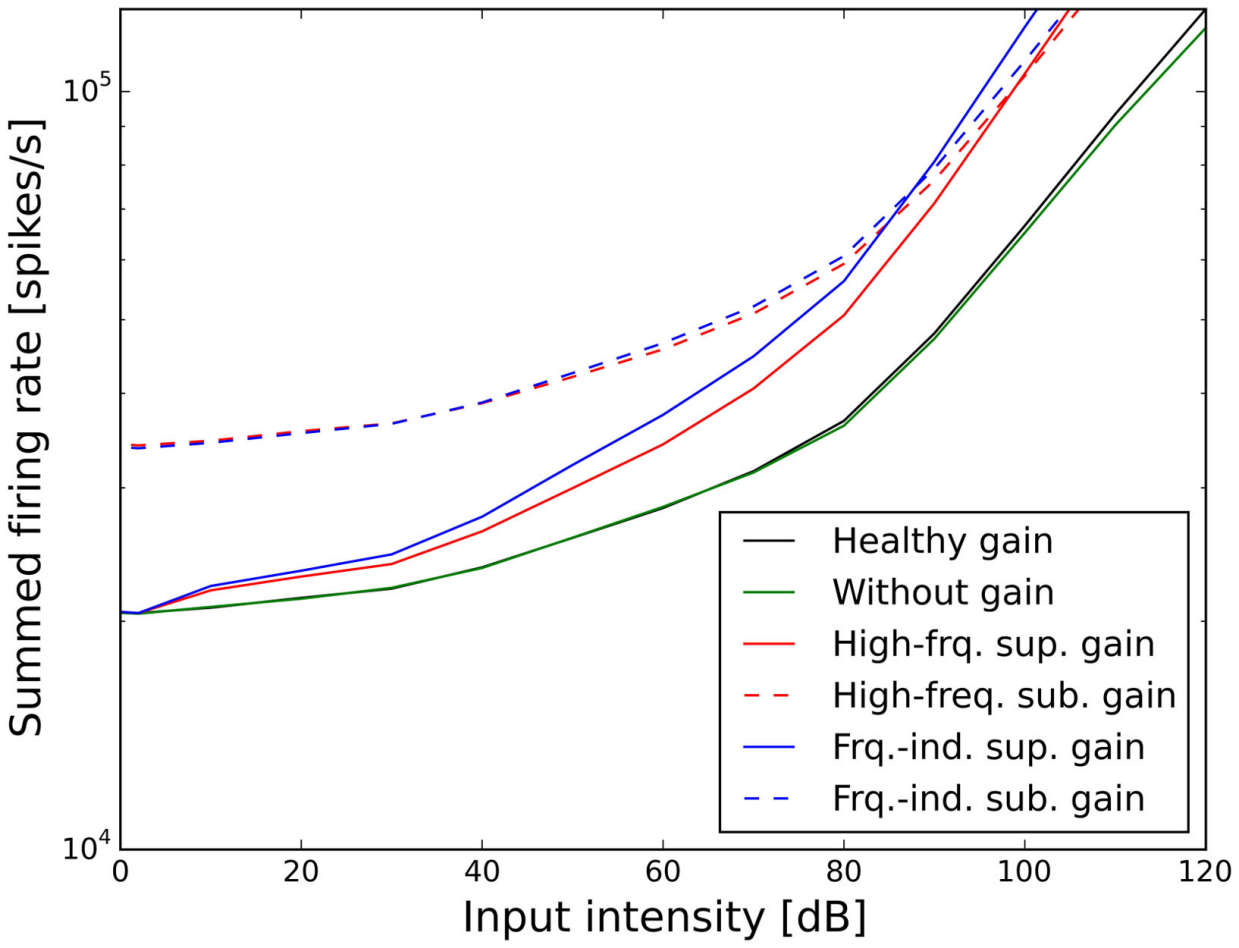
**Effect of the different scenarios with linear gain change on total neural activity in the model**. Results for stimulation with 1 kHz tones are shown.

Figures 7 and 8 show the ELCs and the growth functions of neuronal activity for all possible variants of linear gain mechanisms. Both the supra-threshold frequency-independent and the supra-threshold frequency-specific gain caused a very strong increase in the growth of neural activity as soon as sound intensity exceeded the HT (solid lines in Figure 8). This increase was also reflected in the ELCs in Figures 7A,B, where the 2-, 10-, 20-, and 30-phon ELCs were very close together, indicating that already slight increases in the intensity of the presented tone resulted in a disproportionally large, almost step-like increase in the perceived loudness. However, besides the strong loudness growth for low intensities, both supra-threshold linear gains fit the hyperacusis patient data reasonably well, with the only notable deviations occurring at 125 Hz and 8 kHz, similar to the results obtained for the supra-threshold gain mechanisms (see above). In contrast, both sub-threshold linear gains amplified spontaneous activity levels to such a degree that the 80-phon ELC was below the HT line of the patients. This means that even in the absence of a stimulus, neural activity was as high as for stimulation with a 1-kHz tone at 80 dB SPL in the normal model. As this would correspond to the perception of an 80-phon sound, it could be interpreted as a tinnitus that is as loud as 80 phon. Due to the constant presence of this 80-phon-tinnitus neural activity, the 2- to 80-phon lines accumulated below the HT (Figures 7C,D), and the activity growth curves showed a strong offset (dashed lines in Figure 8). Interestingly, loudness growth above the “tinnitus intensity” seemed to be much shallower than loudness growth functions in hyperacusis patients (12, 13).

In the case of the sub-threshold high-frequency linear gain, the model predicted a high-frequency tinnitus, reminiscent of the results obtained with other models of tinnitus generation [e.g., Ref. (26, 29)], albeit probably at unrealistically high volume. The sub-threshold frequency-independent gain on the other hand produced phantom sounds with broad-band sound characteristics, which are typically not observed in hyperacusis patients.

## Discussion

In this study, we have used a computational model of the ear and AN in conjunction with a gain stage with adjustable response gain to model loudness perception and changes in auditory gain that might underlie hyperacusis. After initial tuning, the model provided a very good fit to standard functions for loudness in sones, and to loudness discomfort levels of normal-hearing subjects. We then evaluated how well different types of auditory gain changes would be able to account for LDL data from hyperacusis patient data with normal HTs. Our main finding is that a power-law gain that amplified only sound-evoked responses and not spontaneous neuronal activity provided the best fit to the data, whereas increases in linear gain and gain increases that also amplified spontaneous activity did not give plausible results.

The AN model that we employed in this study is based on an established model by Meddis and co-workers, which reproduces salient features of cochlear and AN physiology (36, 38, 39). For computational efficiency of the simulations, we simplified the hair cell and AN model based on the implementation in BRIAN.hears (41), and adjusted parameters such that fibers with high, medium, and low spontaneous firing rates were generated. While our simplifications might have consequences for temporal response patterns, they should not affect the relation between sound intensity and overall AN activity. This relation is important for our model, since a core assumption is that perceived loudness is proportional to the summed activity of all AN fibers. The resulting model produced an exceptionally good fit for the ISO standard for loudness in sones (Figures 3B,C). In order to also produce ELCs that matched the LDL curve of our normal-hearing control subjects, a frequency-dependent weighting factor for the AN activity was required (Figure 4B), even though the model AN activity patterns (Figure 3A) were qualitatively similar to cochlear excitation patterns in classical loudness models (34). We do not know if this is a particular feature of our model implementation, or whether such a tuning step might be also required for other physiological models of AN responses. For our results on hyperacusis, the most important features of the model are the shapes of tuning curves and rate-level functions of AN fibers, and therefore, similar results could also have been obtained with a much less detailed phenomenological model. Whether a more or less detailed model might be best suited for future studies will ultimately depend on the level of detail of data sets on hyperacusis that might become available.

Our efforts of modeling hyperacusis focused on reproducing the pattern of decreased LDLs seen in hyperacusis patients (1, 5), since decreased LDLs are one of the most prominent and best characterized features of the hyperacusis phenotype. Ideally, such data would have been complemented by physiological data in order to derive a detailed model of hyperacusis. Unfortunately, there is almost no physiological data on hyperacusis. A recent fMRI study has reported hyperacusis-related changes in sound-evoked responses along the auditory pathway (47), but this study has been conducted on tinnitus patients, some of which were classified as having a certain degree of hyperacusis/sound level tolerance problems according to LDLs and questionnaire scores. None of the participants had a primary complaint of hyperacusis. Subjects with hyperacusis showed increased activation of the inferior colliculus, medial geniculate body, and auditory cortex for broad-band noise stimuli presented at two different intensities (50 and 70 dB SPL), but the effect of hyperacusis was only significant for 70 dB SPL. Moreover, they showed an inverse correlation between the magnitude of signal change in response to the 70-dB SPL stimulus and the loudness discomfort level. Another recent fMRI study on three hyperacusis patients has indicated that hyperacusis-related changes in brain activation might also comprise non-auditory brain areas, specifically the frontal lobes and parahippocampus (48), probably reflecting discomfort or distress elicited by the sound. However, modeling BOLD responses and non-auditory brain areas was beyond the scope of our study. Ideally, human data on hyperacusis would be complemented by detailed physiological from single neurons or small populations of neurons obtained from animal studies, to enable detailed modeling of the mechanisms. However, the development of animal models of hyperacusis has only just started. Hopefully, future models might successively incorporate neural data on hyperacusis when detailed data sets become available, and we hope that our investigation will provide a good starting point for this.

More than a third of hyperacusis patients show normal HTs (1, 5), and we have therefore concentrated our modeling efforts on this group and utilized a model with normal HTs and no further cochlear damage. However, normal HTs do not necessarily indicate the absence of cochlear damage. In fact, there can be considerable deafferentation of AN fibers, loss of spiral ganglion neurons, or even loss of IHCs while HTs are still normal (15, 16, 18, 20). It is conceivable that such forms of “hidden hearing loss” could be responsible for the development of hyperacusis in patients with normal HTs. In fact, a recent study in mice has demonstrated that noise-induced deafferentation of AN fibers leads to increased amplitudes of the acoustic startle response at moderate sound intensities, which was interpreted as hyperacusis-like behavior (49). However, at high sound intensities, startle amplitudes of the noise-exposed mice were lower than those of control mice, which casts slight shadows of doubt whether the mice actually had hyperacusis. In our model, we therefore chose not to include AN fiber deafferentation or IHC loss. In the context of gain models of hyperacusis, these forms of obscured cochlear damage would make it more difficult to generate hyperacusis, since they reduce AN activity, and thus greater gain increases would be required. The gain increases in our model could therefore be seen as a “conservative estimate” of the changes in physiological responses to and/or perceptual evaluation of AN activity. On the other hand, the neural representation of loudness in the brain will surely involve a range of processing steps and transformations based on the input from the AN, and therefore assigning physiological significance to the numerical value of our gain factors might be rather tenuous.

In our model, a supra-threshold gain mechanism that amplified sound-evoked AN responses, but not spontaneous activity, provided the best fit to the hyperacusis patient data (Figures 5A,B and 7A,B). A similar increase in sound-evoked responses has been recently reported for chopper neurons in the ventral cochlear nucleus of cats after noise-induced hearing loss (50). However, at this point in time, we can only speculate whether this might constitute a neural correlate of a hyperacusis gain, since there are currently no behavioral tests for hyperacusis in animals. Nevertheless, even though the neural mechanisms remain to be determined, our finding that a supra-threshold gain accounts much better for the hyperacusis data than sub-threshold gain immediately distinguishes the putative “hyperacusis gain” from the gain increases in current tinnitus models, where the gain increase amplifies spontaneous activity (26–30). For a tinnitus model, amplification of spontaneous activity is a crucial feature, since the resulting increase in spontaneous activity in the central auditory system is generally regarded as a neural correlate of tinnitus (19, 51). However, when such a “tinnitus gain mechanism” (sub-threshold gain) was employed in our simulations to model the decrease in LDLs seen in hyperacusis patient data (Figures 5C,D and 7C,D), the resulting loudness growth was inconsistent with loudness growth functions reported for hyperacusis patients (12, 13). Moreover, in the case of an increase in linear gain, both frequency-independent and frequency-dependent sub-threshold gain produced tinnitus with a loudness of around 80 phon, with broad-band sound characteristics for the frequency-independent and a high-pitched sound for the frequency-dependent gain increase (Figures 7C,D), which is rather unlikely, especially for the broad-band case. Therefore, with the sub-threshold or “tinnitus-type” gain, it seems very difficult to reproduce the hyperacusis pattern of decreased LDLs without causing unrealistic “side-effects” at low sound intensities. On the other hand, any type of hyperacusis gain would amplify any tinnitus-related neural activity (also if it is caused by a different mechanism), which would greatly facilitate the detection of tinnitus even if the initial tinnitus signal is just a very slight elevation of neuronal activity. This could explain why tinnitus is so common in hyperacusis (1), 107 out of the 134 hyperacusis patients with normal hearing thresholds in our data set also perceived tinnitus, whereas the majority of tinnitus patients do not experience hyperacusis (4, 7). Interestingly, tinnitus patients with hyperacusis might perceive their tinnitus as louder than those without hyperacusis (52), which would also be consistent with the scenario of a hyperacusis gain amplifying tinnitus-related neural activity.

In our simulations, a power-law gain increase provided good fits to hyperacusis patient data (Figure 5), whereas the predictions obtained for the linear gain were generally not consistent with the data (Figure 7). Our findings are thus in good accordance with the results of a recently proposed phenomenological model, which predicted that hyperacusis could be due to an increase in non-linear gain in the auditory system (25). Our simulations now show that this prediction also holds when a model based on simulations of the cochlea and AN is applied to patient data, which had not been attempted in the previous study. We currently do not know how such a change in power-law gain or non-linear gain might be implemented in the brain. It could possibly be achieved by altering the balance of excitation and inhibition in a recurrent network, for example at the level of the auditory cortex. Another candidate might be cortical reorganization, where high-frequency neurons become more responsive to low frequencies, and thereby increase the bandwidth of their tuning after cochlear damage (53). Cortical reorganization could increase the number of neurons responding to sound, thus increasing perceived loudness and potentially leading to hyperacusis symptoms. Interestingly, cortical reorganization has been observed in animals studies when the increase in HT after noise exposure exceeded 20 dB (54), thus already for relatively mild hearing loss, not too different from the average HT increase seen in hyperacusis patients in the high-frequency range (1, 5). It would thus been very interesting to investigate whether hyperacusis patients also show signs of cortical reorganization.

Hyperacusis-related changes in neural processing at a higher level of the auditory system would also be consistent with recent results on the effects of monaural earplug-induced auditory deprivation on loudness perception and acoustic reflex thresholds. While acoustic reflex thresholds (a reflex arc that involves the brainstem) changed in opposite directions for the plugged and the unplugged ear, perceived loudness was increased for both ears, suggesting a gain change at a higher processing stage of the auditory system that integrates input from both ears (22). A recent imaging study has found that the inferior colliculus, the medial geniculate body as well as the auditory cortex showed enhanced activity in subjects with sound tolerance problems (47). Whether increased activation at a sub-cortical level is due to increased neuronal gain at this stage, or rather due to feedback from higher brain areas, remains to be determined.

In our model, both gain increases in the form of a “master volume control” (frequency-independent gain increase), and a frequency-specific gain increase in the high-frequency range, provided a good fit to the hyperacusis LDL data, with the exception of 125 Hz and 8 kHz, where LDLs were predicted mostly too high (Figures 5A,B and 7A,B). Allowing more degrees of freedom for the frequency-specific gain could have improved the fit to the data here. However, for these frequencies, the highest output levels of the equipment used to measure LDLs in the study by Sheldrake et al. (5) were 90 and 100 dB HL, respectively, compared to 120 dB HL for all other frequencies (except 250 Hz for which the output limit was 110 dB HL), which might have skewed the average LDLs, and we therefore chose not to introduce additional parameters to improve the fit there. The more important point of the results is that both the frequency-independent and the frequency-dependent gain provided comparable results. Even though this result seems surprising at first, it can be easily explained by the fact that low-frequency tones can activate AN fibers along the whole length of the cochlea when presented at sufficient volume. In chinchillas, for example, AN fibers with best frequencies in the range of 8–16 kHz can be activated by 30 Hz tones presented at 80–100 dB SPL (55), and therefore, gain increases in the high-frequency range can also increase the overall neural activity evoked by low-frequency tones. The model simulations therefore indicate that both scenarios could in principle be candidates for the mechanism underlying decreased sound tolerance in hyperacusis. However, while both mechanisms provided a good fit for the average pattern of LDLs of hyperacusis patients, the high-frequency mechanism would struggle to account for LDLs as low as 50 or even 40 dB HL at low frequencies, which are observed in a significant fraction of patients (5). Moreover, frequency-specific vs. non-specific changes in gain could also potentially signify different kinds of cochlear damage as the underlying cause. On the one hand, noise-induced damage usually affects the high-frequency range more strongly than the low-frequency range (15, 56, 57), and could therefore lead to gain increases in the high-frequency channels. On the other hand, age-related changes like degeneration of spiral ganglion neurons (18), and also hair cell loss due to ototoxic drugs (20) can affect all parts of the cochlea, and could therefore trigger gain changes similar to our master volume control. In studies with earplug-induced deprivation, increases in perceived loudness have been observed also at frequencies where the earplug did not provide attenuation (21) and even in the unplugged ear (22). These results might be best explained through changes in a gain mechanism similar to our frequency-independent master volume control acting across all frequencies and possibly also across ears (note that we did not model integration across ears, as the LDL measurements on hyperacusis patients were only conducted monaurally via headphones). Furthermore, the earplug results suggest that the evaluation of loudness in the brain might occur at a rather high level of the auditory system by pooling across frequencies and ears. Dysfunction of such a mechanism would produce equal changes across frequencies and thus account for the typical hyperacusis phenotype. Finally, another indication that a frequency-independent mechanism might underlie hyperacusis comes from a study where hyperacusis patients were treated with acoustic stimulation in the high-frequency range, which caused a decrease of perceived loudness even at low frequencies that were not stimulated (13). Therefore, the frequency-independent increase in gain might be a more likely candidate as the mechanism underlying hyperacusis. We hope that our modeling results will inspire and guide further experimental studies on hyperacusis that will help unravel the physiological basis of abnormal loudness perception and lead to improved treatments.

## Conflict of Interest Statement

The authors declare that the research was conducted in the absence of any commercial or financial relationships that could be construed as a potential conflict of interest.
